# Molecular mechanisms and applications of antimicrobial secondary metabolites of *Bacillus subtilis* based on biofilm and quorum sensing

**DOI:** 10.1128/aem.00241-26

**Published:** 2026-05-21

**Authors:** Jun Feng, Hongyu Luo, Zejia Zhang, Zelin Wang, Mingxuan Wang

**Affiliations:** 1 Department of Food Science and Engineering, School of Health Science and Engineering, University of Shanghai for Science and Technology 47863, Shanghai, China; The Pennsylvania State University, University Park, Pennsylvania, USA

**Keywords:** *Bacillus subtilis*, secondary metabolites, biofilm, quorum sensing, application

## Abstract

Antimicrobial resistance remains a significant global threat to human health, but microorganisms have long been a crucial source of novel antibiotics. The widely distributed gram-positive bacterium *Bacillus subtilis* produces an abundance of secondary metabolites, and their antibacterial activities could have significant applications in food, agriculture, and aquaculture areas. These secondary metabolites exert antibacterial effects through mechanisms such as microbial cell membrane structure disruption, cell wall synthesis interference, and cellular metabolic activity inhibition. In contrast to microorganisms such as *Streptomyces*,*B. subtilis* forms characteristic biofilms and exhibits quorum sensing, which play important roles in the production of secondary metabolites and their antimicrobial effects. However, limited attention has been focused on the unique molecular mechanisms associated with biofilms and quorum sensing. In this review, we first summarize the typical secondary metabolites produced by *B. subtilis*. We then mainly focus on the molecular mechanisms associated with the regulation of biofilms and quorum sensing by antimicrobial secondary metabolites, and the effects of biofilms and quorum sensing on the biosynthesis of antimicrobial secondary metabolites. The applications of antimicrobial secondary metabolites in the fields of food, agriculture, and fisheries, based on the regulation of biofilm and quorum sensing, are also summarized. Finally, we highlight the need for further research into the regulatory networks related to biofilms, quorum sensing, and metabolites to facilitate a deeper understanding of the antimicrobial properties of *B. subtilis*, which may provide theoretical support for the development of novel antimicrobial food technologies.

## INTRODUCTION

Antimicrobial resistance (AMR) remains a significant global threat to human health. In recent years, the excessive use of antibiotics has led to continuous increases in the number of antibiotic-resistant bacteria (1). The World Health Organization predicts that without new effective treatment strategies, the annual global death toll due to AMR could rise from at least 700,000 to 10 million by 2050 (2). Therefore, there is an urgent need to develop novel antibiotics to address the escalating problem of antibiotic resistance (3).

Microorganisms have long been a crucial source of novel antibiotics. Among the many microbes that produce antibiotics, actinomycetes, particularly those in the genus *Streptomyces*, have garnered significant attention due to their ability to synthesize diverse bioactive secondary metabolites, including antibiotics (4). Extensive research has allowed the generation of many of these compounds to reach scales suitable for industrial production (5–8). However, the exclusive screening of actinomycetes for new antibiotics has become increasingly challenging (9), necessitating the exploration of novel antibiotic sources from a broader range of suitable microorganisms.

*Bacillus subtilis* is a non-pathogenicgram-positive bacterium that is capable of forming stress-resistant endospores. *B. subtilis* exhibits rich physiological diversity and is widely distributed in plant tissues, soil, food, and the intestinal microbiota of some animals. It is non-toxic to humans, animals, and the environment and has remarkable potential in terms of the synthesis of bioactive compounds and genetic diversity (10). Due to its well-developed secretion system, *B. subtilis* produces various antimicrobial metabolites, including non-ribosomal peptides (e.g., surfactin, fengycin, and iturin), ribosomal peptides, polyketides, and volatile compounds (11), which are currently widely applied in fermented food production (12). In contrast to other microorganisms, such as *Streptomyces*, *B. subtilis* can form characteristic biofilms and exhibits quorum sensing. Antimicrobial secondary metabolites and biofilm formation/quorum sensing can effectively regulate each other, but the mechanisms involved and their interactions have received limited attention. Biofilms are surface-associated microbial communities encased in an extracellular matrix (13) and are considered the primary survival mode for bacteria in natural environments (14).*B. subtilis* has the ability to switch from a motile state to a sessile state and form a biofilm, and it has been widely used in biofilm research (15). Quorum sensing is commonly employed by bacteria as an intercellular communication mechanism to regulate the expression of genes involved in multiple cellular processes, including virulence factors, sporulation, motility, toxin production, and biofilm formation (16). Studies also suggest that *B. subtilis* PS-216 may utilize quorum sensing to modulate its motility and biofilm construction processes (17).

Many reviews have summarized the diversity, biosynthesis, and antimicrobial activities of secondary metabolites produced by *Bacillus subtilis*, as well as the molecular mechanisms underlying biofilm formation or quorum sensing, individually. However, in these reviews, biofilm formation and quorum sensing are primarily treated either as regulatory backgrounds or as independent physiological traits, rather than being integrated into a unified framework that explains how antimicrobial secondary metabolites both regulate and are regulated by these multicellular behaviors (11, 18–20).

In contrast, the present review focuses on the bidirectional and dynamic interplay among antimicrobial secondary metabolites, biofilm formation, and quorum sensing in *B. subtilis*. We first summarize the antibacterial substances produced by *B. subtilis* and the mechanisms involved in their synthesis. We then mainly focus on analyzing the mechanisms that regulate antibacterial metabolites from the perspectives of how antibacterial metabolites regulate biofilms and quorum sensing, as well as how biofilms and quorum sensing further influence antibacterial effects. Finally, the applications of these antibacterial metabolites in the food and agricultural fields are reviewed. Investigating the unique regulatory networks related to biofilms, quorum sensing, and metabolites, as well as the stability and safety of antibacterial substances in large-scale applications, may provide theoretical support for the development of novel food antibacterial technologies.

## TYPICAL ANTIBACTERIAL SECONDARY METABOLITES

Antibacterial peptides produced by *B. subtilis* can be classified as non-ribosomally synthesized peptides and ribosomally synthesized peptides based on their biosynthetic pathways. Polyketides and various volatile compounds are two other major classes of antibacterial compounds produced by *B. subtilis* (Table 1).

**TABLE 1:** Typical antibacterial substances produced by *B. subtilis*

Type	Compound	Structural formula	Reference
Non-ribosomal peptides	SurfactinC15	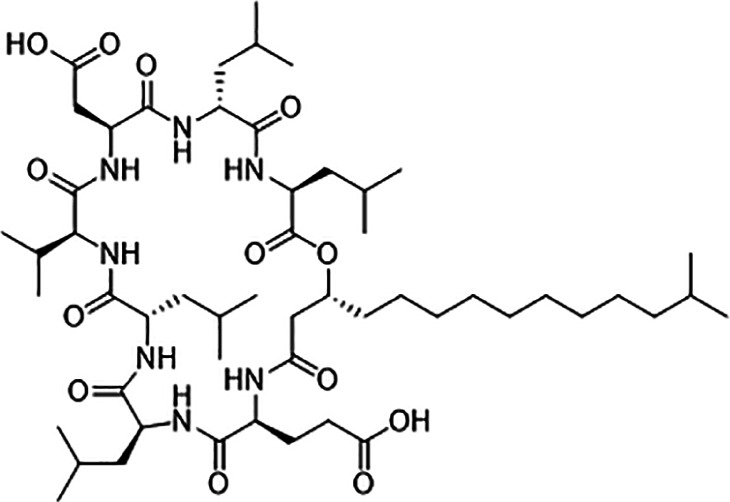	(10)
	Fengycin B	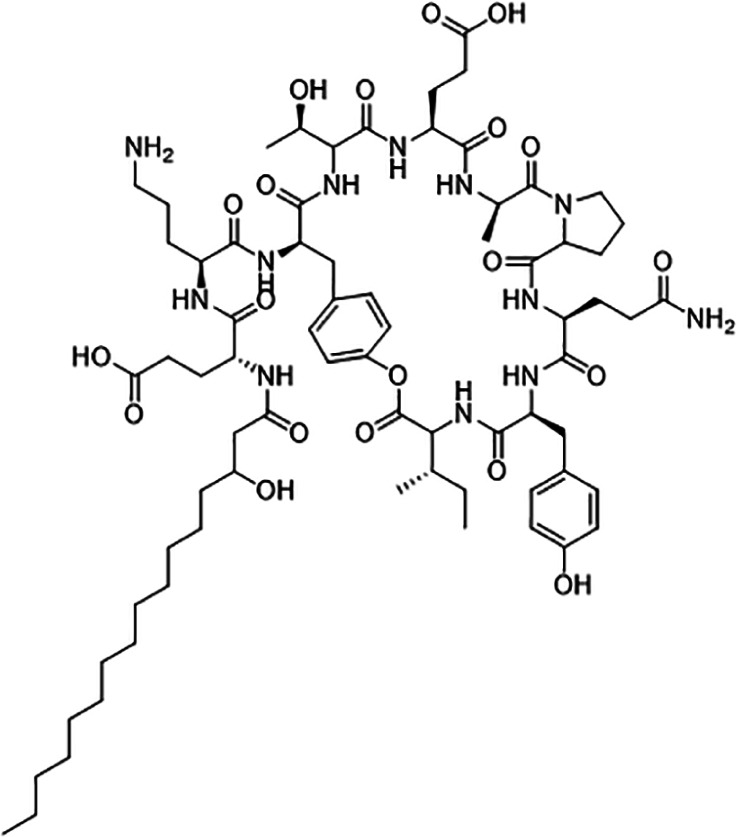	(21)
	Iturin A	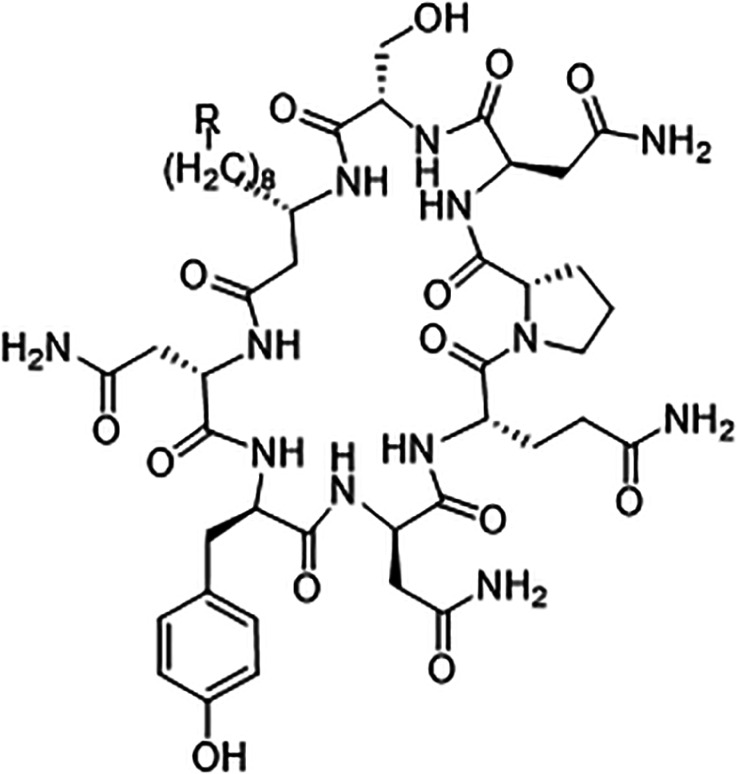	(22)
Ribosomal peptides	Mersacidin	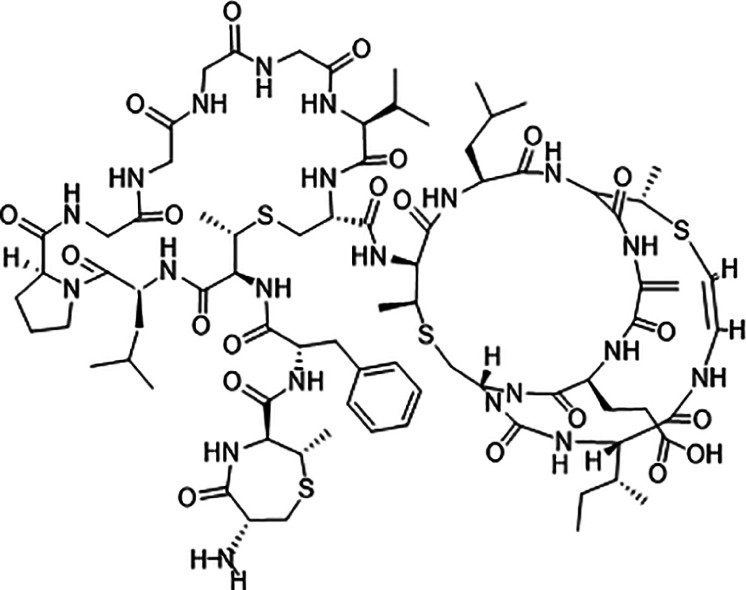	(23)
Polyketides	Macrolactin A	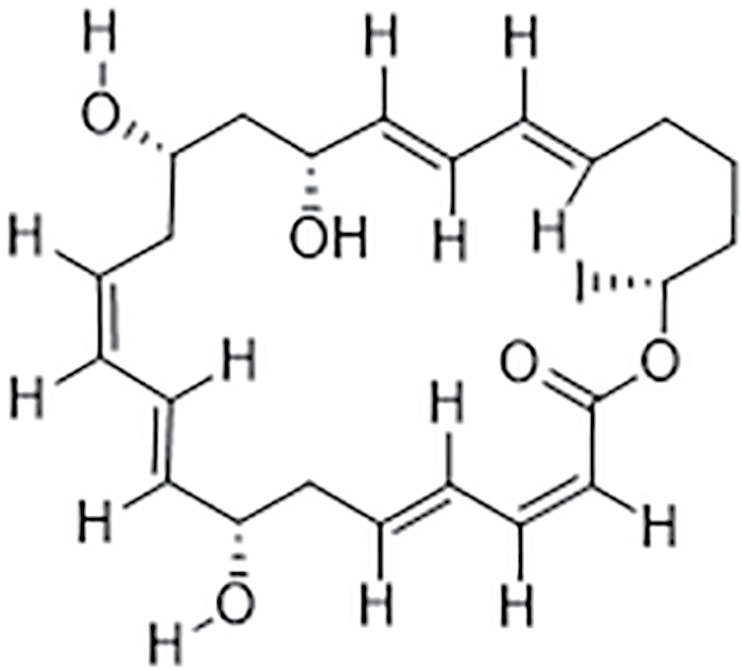	(24)
	Difficidin	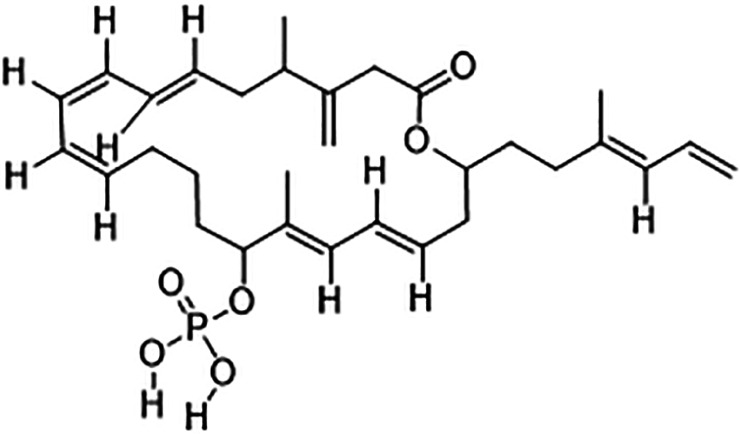	(25)
	Bacillaene A	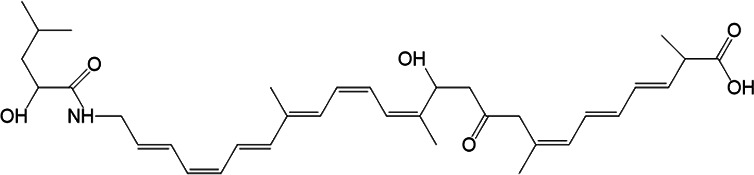	(26)

### Non-ribosomalpeptides

Lipopeptides are important non-ribosomal peptides in *B. subtilis*, and they mainly include surfactin, fengycin, and iturin. In addition to lipopeptides, *B. subtilis* can produce various cyclic peptide-type and linear peptide-type non-ribosomal peptides, where the former are formed of several amino acid residues connected end-to-end via peptide bonds to form a cyclic structure, and the latter are composed of multiple amino acid residues arranged linearly. These structures are relatively simple, but they still have antibacterial activities.

#### Surfactin

In 1968, surfactin was first isolated from *B. subtilis* culture supernatant (10). Surfactin is a lipopeptide primarily produced by the gram-positive bacterium *B. subtilis* and a representative biosurfactant in the cyclic lipopeptide family, which is synthesized by non-ribosomal peptide synthetases and transported extracellularly (27). The unique cyclic structure of surfactin consists of hydrophobic β-hydroxy fatty acid chains with various lengths linked to a heptapeptide ring and enclosed by a lactone bridge between the β-hydroxy functional group of the fatty acid and C-terminal of the heptapeptide (28). The amphiphilic nature of surfactin explains its extremely strong surface activity (29), and it is one of the most potent known biosurfactants. The diverse biological activities of surfactin include antiviral, antitumor, antibacterial, anticoagulant, and cholesterol-lowering effects (11). Its antibacterial activity is particularly remarkable, and it can penetrate the cell membranes of various bacteria. Moreover, surfactin can prevent the adhesion of different pathogens to the surfaces of objects, as well as destroying the integrity of cell membranes by inserting into the lipid membrane to increase the cell membrane’s permeability, thereby interfering with the barrier function of the membrane (30) to cause antibacterial and hemolytic effects. These characteristics of surfactin have attracted significant attention due to its possible applications in food, agriculture, medicine, and other fields (31). Surfactin also interacts with cell membrane surface proteins to interfere with transmembrane ion transport and the functions of vital enzymes in cells (32). Studies have shown that treating *Staphylococcus aureus* with surfactin creates an ion concentration gradient across the cell membrane to generate a membrane potential, which plays a key role in energy transfer and nutrient absorption, and decreasing the membrane potential inhibits cellular metabolic activities (33).

#### Fengycin

Fengycin exhibits remarkable broad-spectrum antifungal activity. The peptide chain of fengycin contains four D-amino acids and ornithine, and it specifically inhibits the growth of filamentous fungi (34, 35). Fengycin has a β-hydroxy fatty acid chain with 16–19 carbon atoms, and it exists in two main forms that differ by one amino acid residue (36): D-Ala in fengycin A and D-Val in fengycin B. Fengycin is synthesized by non-ribosomal peptide synthetases, and five genes (fenA to fenE) encode the synthetase (21).

#### Iturin

Members of the iturin family, including iturin and bacillomycin, exhibit potent antifungal activity and contain seven α-amino acids and a β-amino fatty acid (37). In particular, iturin A has remarkable antifungal properties and minimal toxicity, making it highly promising for biopesticide development and fungal disease treatment (22). Many strains of *B. subtilis* can synthesize members of the iturin family, and their biosynthesis in *B.subtilis* species is regulated by operons (24).Similar to the mechanism associated with most lipopeptides, iturin binds to specific lipid components in fungal cell membranes, embeds into the membrane, and forms ion channels, leading to the leakage of intracellular ions and small molecules, thereby inducing cell death and inhibiting fungal growth (38). Iturin A significantly alters membrane fluidity and disrupts the stability and structure of membranes by acting on dielaidoylphosphatidylethanolamine molecules (39). In addition, iturin can disrupt membrane integrity by interacting with sterols (e.g., ergosterol) in target cell membranes or with phospholipids at high concentrations (40). In food production, iturin is commonly used to produce emulsions, inhibit fat globule aggregation, improve the texture of products, and extend shelf life (22).

### Ribosomal peptides

Ribosomal peptides, also known as ribosomally synthesized and post-translationally modified peptides (RIPPs), are typically derived from short precursors that undergo post-translational modifications to form mature compounds (41). Compared with non-ribosomal antibiotics, RIPPs are more amenable to genetic manipulation to enhance their antibacterial activity (42). Members of this family mainly include antibiotics and antifungal proteins, such as subtilin (43).

#### Lantibiotics

Lantibiotics are antibacterial peptides produced by bacteria containing diverse amino acid residues. Based on their structural characteristics, lantibiotics are classified into type A and type B, where type A lantibiotics have 21–38 amino acid residues and prominent linear secondary structures. They induce death in gram-positive target cells by forming voltage-responsive pores in the cytoplasmic membrane (44). Subtilin, a 32-amino acid pentacyclic lantibiotic containing lanthionine, inhibits bacterial growth by binding to peptidoglycan (a bacterial cell wall precursor) and interfering with cell wall synthesis (45). An endophytic *B. subtilis* strain BSn5 stimulates plant defense responses through the production of the lantibiotic subtilin combined with flagellin (46). By contrast, the type B lantibiotic mersacidin has a globular structure and inhibits cell wall biosynthesis by binding to lipid II (23). Sublancin, a natural product of *B. subtilis* 168, features a β-methyl lanthionine bridge and two disulfide bonds, conferring high stability (47). Similar to subtilin, sublancin has antibacterial activity against a spectrum of gram-positive bacteria and inhibits bacterial spore growth (48).

### Polyketides

Polyketides are a class of bioactive compounds produced by microorganisms and well known for their broad-spectrum activities in plant disease control and medicine, including antibacterial, immunosuppressive, and various antagonistic effects (49). For example, polyketides are synthesized via decarboxylative Claisen condensation of extender units (e.g., malonyl derivatives) with polyketide chains (50) to generate enzyme-bound β-ketoacyl intermediates (51) as the fundamental step in polyketide chain assembly. Each condensation reaction elongates the polyketide chain. The polyketide synthase multienzyme system is central to polyketide biosynthesis and enhances structural diversity. Type I polyketide synthases are modular megasynthetases, where each module typically contains indispensable domains: β-ketoacyl synthase, acyltransferase, and acyl carrier protein. Acyltransferase selects and loads the correct acyl substrate onto the acyl carrier protein, which carries the growing polyketide chain and shuttles substrates between enzyme domains to ensure ordered synthesis (52). Bacillaene is a polyketide antibiotic produced by *B. subtilis*, synthesized by a large hybrid PKS/NRPS enzyme complex encoded by the *pks* gene cluster. It primarily exhibits antibacterial activity by inhibiting protein synthesis in competing bacteria, thereby contributing to the ecological competitiveness of *B. subtilis* within microbial communities (53).

Macrolides are typical antibacterial polyketides that interfere with bacterial membrane function, disrupt normal cellular physiology, and inhibit gram-positive/negative bacteria and fungi (54). Macrolactins are 24-membered macrolides produced primarily by marine microorganisms, with potent antibacterial activities against *S. aureus*, *B. subtilis*, and *Escherichia coli* (55). Macrolactin A suppresses plant pathogens and related diseases, such as *Fusarium oxysporum* and *Rhizoctonia solani*, which are pathogens that attack diverse crops and cause infections (24). Difficidin is a polyene polyketide encoded by type I polyketide synthase in the DIF operon and has been confirmed to inhibit bacterial pathogens (25), including those causing tomato bacterial wilt (56) and fire blight (57).

### Volatile organiccompounds

Volatile organic compounds produced by *B. subtilis* are low-molecular-weight compounds with antibacterial activity (58). Volatile organic compounds have often been used as biocontrol agents to reduce postharvest losses in fruit and vegetable crops (59) because they can inhibit mycelial growth and the germination of spores produced by various pathogenic fungi (60). According to previous research, the volatile organic compounds produced by *B. subtilis* CF-3 disrupt hyphal morphology, exhibit potent antibacterial effects, and inhibit pigment production in pathogenic bacteria (61). Volatile organic compounds produced by plant growth-promoting rhizobacteria hold promise for inhibiting plant pathogens, promoting plant growth, and inducing systemic disease resistance in plants (62). The volatile compound 2,3-butanedione affects ergosterol biosynthesis. Ergosterol is a component of fungal cytoplasmic membranes that binds to phospholipids to form stable phospholipid phases, thereby enhancing the stability of fungal membranes. Disrupting ergosterol biosynthesis leads to membrane damage and imbalanced cell permeability (63).

## REGULATION OF BIOFILMS AND QUORUM SENSING BY ANTIBACTERIAL SECONDARY METABOLITES

### Biofilms and mechanisms

#### Formation and structuralcharacteristics of biofilms

The formation of *B. subtilis* biofilms is a dynamic and ordered process. In response to external signals such as nutrient depletion, hypoxic environments, or surface attachment, cells actively adjust gene expression levels to reduce the activity of flagellar genes while enhancing the expression of extracellular matrix synthesis genes, thereby switching from a planktonic state to a sessile state and initiating biofilm construction (14). During this process, cells begin secreting extracellular polymeric substances (EPS), proteins, nucleic acids, and other materials, gradually forming an extracellular matrix framework (64). EPS is crucial for biofilm formation, structural stability, and protecting cells against environmental stresses. Biofilms aggregate within these EPS matrices and use EPS as adhesive elements to attach to specific surfaces (65). Biofilms can be classified into different types based on the culture conditions, such as floating biofilms that form at the air–liquid interface, colony biofilms at the air–solid interface, and surface-adherent biofilms submerged at the solid–liquid interface. Mature biofilms generally exhibit highly heterogeneous structures, providing a suitable microenvironment for the survival of microbial communities (66), and this heterogeneity is also closely associated with the synthesis and action of antibacterial secondary metabolites.

##### EPS

Biofilm formation is regulated by multiple genes in *B. subtilis*. EPS are the primary components of biofilms and are synthesized via the EPS synthesis proteins encoded by the *epsA–O* operon. Proteins encoded by this operon play key roles in EPS synthesis, forming complex colony structures and films. For example, EpsA-O is the main extracellular polysaccharide in *B. subtilis* and is synthesized by a cluster of 15 gene products of the *epsA–O* operon. EpsA and EpsB regulate tyrosine kinase synthesis to control EPS production (67); EpsE acts as a glycosyltransferase to regulate polysaccharide secretion (68); and EpsC, EpsM, and others dominate the synthesis of N,N′-diacetylbacillosamine, which is a unique modified monosaccharide synthesized by only a few bacterial species (69). These proteins collaboratively construct the basic skeleton of biofilms, enabling stable adhesion and aggregation of bacterial cells.

##### Protein components

TasA is the main component of the biofilm matrix, and it was reported to form functional amyloid fibers contributing to biofilm structure and stability. However, recent electron cryomicroscopy structures of TasA fibers showed that rather than forming amyloid fibrils, TasA monomers assemble into fibers through donor-strand exchange, with each subunit donating a β-strand to complete the fold of the next subunit along the fiber (70). TasA influences cell physiology and gene expression, such as by stimulating motility gene expression, downregulating matrix expression to promote colony dispersion, and interacting with cell membranes to affect membrane fluidity (15). TasA also exhibits broad-spectrum antibacterial activity (71). TasA is expressed as part of the *TapA-sipW-tasA* operon; the operon further encodes two accessory proteins, TapA and SipW. The signal peptidase SipW processes these proteins into mature forms (64). Regarding TapA, genetic and biochemical studies demonstrate that it is required for efficient TasA fiber formation *in vivo*, but not necessarily as a structural matrix component itself (72). Rather, TapA appears to function in a chaperone-like capacity, facilitating the correct assembly and stabilization of TasA fibers during biofilm development. This observation suggests that TapA’s primary function is not to serve as a static scaffold or anchor but instead to promote or nucleate TasA polymerization (73). Consistent with this, recent work supports a model in which TapA contributes to strand complementation or donor-strand exchange during TasA fiber assembly, thereby enhancing fiber integrity and robustness within the extracellular matrix (70). Studies have shown that TapA1-57 is a key component of the functional form of TapA *in vivo*, allowing rugose biofilm architecture to manifest (74). BslA is a hydrophobin protein with an immunoglobulin-like fold, and it has critical roles in bacterial sliding motility and biofilm architecture (75). BslA controls the non-wetting properties and overall architecture of biofilms (76), and it is secreted in the late maturation stage to form a hydrophobic protective layer on the biofilm’s surface. This layer effectively blocks gas and liquid penetration (77), protecting bacteria from external chemical attacks. Through the analysis of the crystal structure of BslA, BslA consists of an Ig-type fold with the addition of an unusual, extremely hydrophobic “cap” region. The central hydrophobic residues within the cap region are crucial for the formation of a hydrophobic, non-wetting biofilm, as they regulate the surface activity of the BslA protein (78).

### Self-regulatory pathways for biofilms

The production of biofilm matrix macromolecules involves a sophisticated genetic regulatory system, where two key transcription factors, i.e., Spo0A and DegU, control the switch for macromolecule synthesis (79).

**Fig 1:**
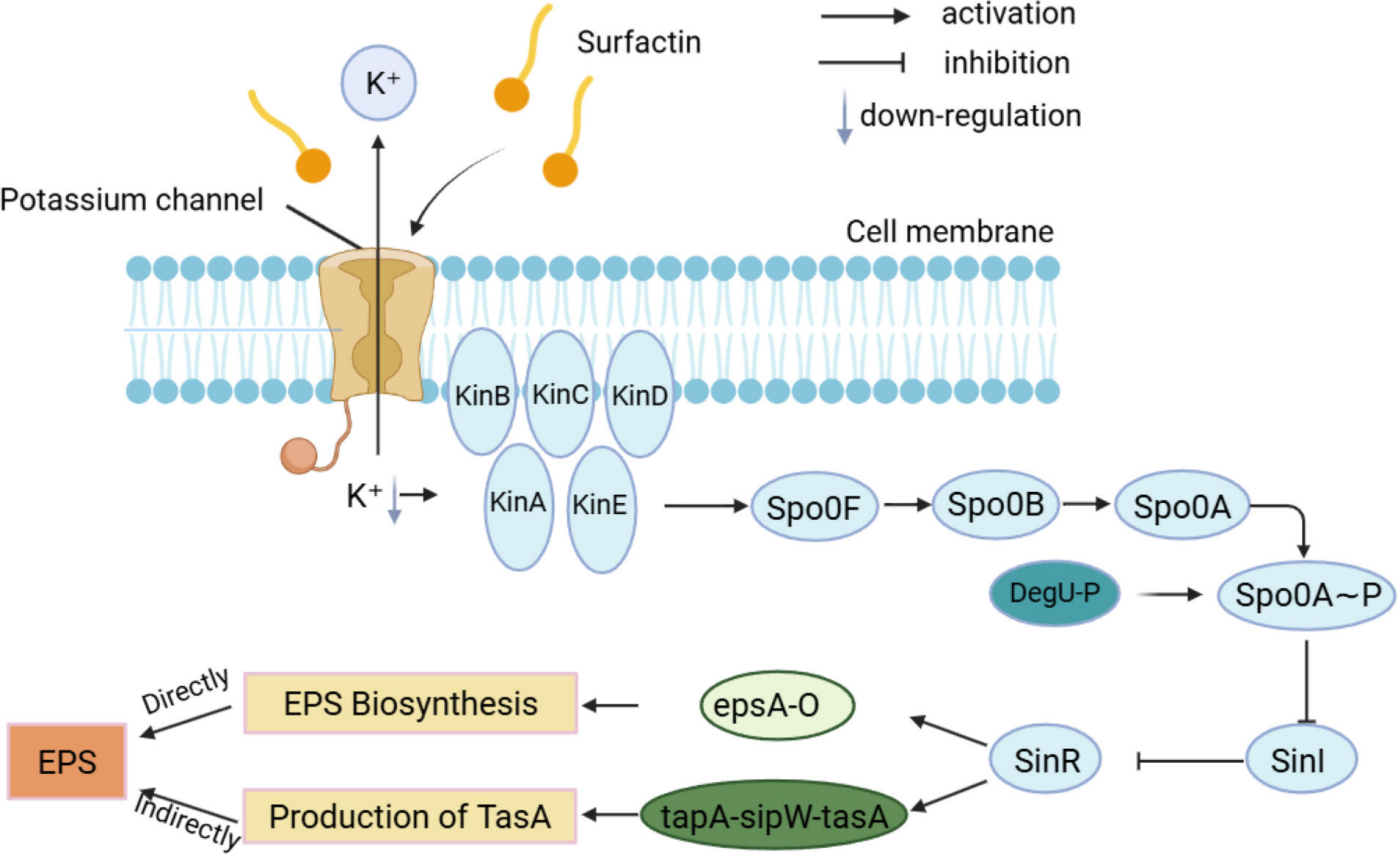
Mechanisms of action of antimicrobial secondary metabolites against biofilms. Surfactin induces pore formation in the cell membrane, leading to potassium ion leakage, which activates histidine kinases KinA*–*E to regulate the phosphorylation of Spo0A via Spo0F and Spo0B. Activated Spo0A (Spo0A∼P) at low levels activates SinI, which inhibits the repressor SinR. Derepression of the *tapA–sipW–tasA* and *epsA–O* operons ultimately regulates the transcription of genes involved in biofilm synthesis.

1. Transcription factor Spo0A: Spo0A is a core regulator of biofilm formation, and its activity in biofilm-related gene transcription and matrix production is governed by phosphorylation (80). Moderate phosphorylation promotes the expression of matrix genes for biofilm formation, whereas high levels induce sporulation gene expression (13). The concentration of Spo0A∼P is influenced bykinases such as KinA, KinB, KinC, and KinD. The repressors SinR and AbrB inhibit the transcription of *TapA* and *Eps* promoters. This inhibition is relieved when Spo0A∼P phosphorylation reaches the threshold for biofilm initiation. In particular, the bistable switch SlrR, together with the anti-repressor SinI, sequesters the matrix gene repressor SinR from promoter elements. By contrast, SlrR specifically clears SinR from the *Eps* and *TasA* promoter regions to allow unimpeded transcription. In addition, SlrR suppresses the transcription of autolysin synthesis genes (79, 81) (Fig. 1). Autolysins have been demonstrated to participate in a range of essential cellular processes, including differentiation, cell lysis, cell wall expansion and remodeling, daughter cell separation, competence development, and motility (82).The primary function of autolysins is in peptidoglycan turnover. In *B. subtilis*, peptidoglycan turnover is a highly coordinated process that is tightly coupled to cell growth, elongation, and division and is primarily mediated by a redundant yet functionally specialized set of autolysins (83). Moreover, autolysins are typically associated with planktonic lifestyles, and they inhibit bacterial motility and dispersion to promote biofilm construction.
2. Transcription factor DegU∼P: DegU∼P indirectly controls the frequency of transcription for biofilm matrix biosynthesis genes. DegU∼P regulates expression of the *BslA* operon (14), and increased levels promote elevation of Spo0A∼P (79).

### Quorum sensingsystems and mechanisms

Quorum sensing is a universal communication strategy in bacteria that enables cell-to-cell signaling to precisely regulate various adaptive traits in response to cell density changes (84). In *B. subtilis*, quorum sensing triggers diverse population responses (85).

ComQXPA is a typical quorum sensing system in *B. subtilis* involving four proteins: the secreted signaling molecule ComX, prenyltransferase ComQ, histidine kinase ComP, and response regulator ComA. Using ComX as an autoinducer (86), the system secretes ComX extracellularly via ComQ processing. When extracellular ComX accumulates to a threshold, it activates and phosphorylates the ComP receptor, thereby inducing the phosphorylation of ComA (87), and further regulating the transcription of multiple genes and synthesis of other molecules.

Quorum sensing in *B. subtilis* is also regulated by the Rap protein family and its cognate Phr peptide family. Cytoplasmic Rap proteins regulate signal transduction by inhibiting their cognate response regulators (Spo0F, DegU, or ComA), either through phosphatase activity that promotes dephosphorylation or by directly preventing DNA binding. In turn, Rap activity is antagonized by their specific Phr peptides. These peptides are synthesized as precursor pre-Phr proteins, secreted into the extracellular environment, and processed to yield mature oligopeptides of five to six amino acids. Upon reaching a threshold concentration at high cell density, the mature Phr peptides are reimported into the cell, where they bind to their cognate Rap proteins and induce conformational changes that abolish Rap inhibitory activity (88). Rap proteins exhibit a modular architecture consisting of an N-terminal α-helical domain linked to a C-terminal region composed of six tetratricopeptide repeat (TPR) motifs (89). As bacterial populations increase, accumulating Phr peptides inhibit Rap proteins, prompting response regulators to activate genomic regions responsible for multicellular behaviors (86).

Studies have shown that when co-culturing the ComQXPA system and the Rap–Phr regulatory system, cells capable of secreting autoinducers exhibit stronger quorum sensing responses than non-secreting cells, where this difference is based on self-perception mechanisms rather than regulatory pathways (90).

### Mechanisms of action of antibacterialsecondarymetabolites on biofilms and quorumsensing

The secondary metabolites of *B. subtilis* are closely associated with biofilm formation and quorum sensing. In particular, these metabolites can directly target the synthesis of biofilm-related components, thereby influencing the structure and regulation of biofilms. In addition, antibacterial secondary metabolites can act as signaling molecules to activate quorum sensing systems, initiating cascades of signal transduction pathways that indirectly modulate biofilm architecture and regulation through intercellular communication.

#### Direct mechanisms of action of antimicrobialsecondarymetabolites on biofilms

##### Effects on biofilms produced by *B. subtilis*

Some secondary metabolites can directly affect biofilm structure and development or regulate the transcription and translation of genes encoding biofilm-related extracellular polymers and proteins. They may also interact with proteins to alter their structure and function. For example, high concentrations of Ca^2+^ can inhibit surfactin-mediated reduction of surface tension, and surfactin-induced reduction of surface tension promotes surface expansion behaviors by biofilms, such as colony spreading, sliding, and swarming motility, thereby enhancing the stability and diffusive growth capacity of biofilm structures (91). Surfactin also increases the content of phospholipid membranes in the central region of biofilms, preventing self-membrane permeabilization through mechanisms such as electrostatic repulsion and increased membrane rigidity (30).

##### Effects on biofilms produced by pathogens

In most cases, bacterial biofilms are the primary targets of antimicrobial secondary metabolites with antibacterial activities that resist invasion by foreign pathogens (92). Lipopeptides and certain volatile substances, characterized by unique amphiphilic structures formed by the conjugation of peptide moieties with amino fatty acids or hydroxy fatty acids, can interact with microbial biofilms, leading to cellular leakage and subsequent cell death (93). Lipopeptides produced by *Bacillus licheniformis*, particularly surfactin, may inhibit biofilm formation in pathogens such as *Salmonella typhimurium*, *Escherichia coli*, and *S. aureus* by altering bacterial surface hydrophobicity, impairing flagellar development, and interfering with cell adhesion. Surfactin primarily induces cell lysis in gram-positive bacteria, and it can also disrupt lipid accumulation, create pores in biofilms, penetrate membranes via hydrophobic interactions, and alter the hydrocarbon chain arrangement and membrane thickness, thereby weakening bacterial viability and reducing the foundation for biofilm formation (94). Surfactin treatment inhibits the expression of genes related to extracellular polysaccharide synthesis in *Enterococcus faecalis*, reducing extracellular polysaccharide levels and ultimately impairing biofilm formation (95). Volatile organic compounds produced by *Bacillus amyloliquefaciens* (a species closely related to *B. subtilis*) significantly inhibit biofilm formation in *Ralstonia solanacearum*, the causative agent of tomato bacterial wilt (96). In addition, bioactive volatile compounds from *B. subtilis* can interact with biofilm-forming proteins in *Pseudomonas aeruginosa* through molecular docking, disrupting their normal function (97).

### Indirect mechanismsrelated to the effects of antimicrobialsecondarymetabolites on biofilms via quorumsensing

#### Effects on biofilms produced by *B. subtilis*

Antimicrobial secondary metabolites play crucial roles in maintaining the stability and function of biofilms produced by *B. subtilis*. As a signaling molecule, surfactin can promote swarming motility and induce the expression of genes involved in synthesizing the biofilm extracellular matrix, thereby indirectly inducing biofilm formation. Surfactin can cause pore formation in the cell membrane, leading to potassium ion leakage and the generation of a membrane potential difference (98). This activates autophosphorylating histidine kinases KinA–E, which then phosphorylate and regulate the phosphorylation of Spo0A through Spo0F and Spo0B (99). Activated Spo0A forms Spo0A∼P and low levels of Spo0A∼P activate the anti-repressor SinI, which inhibits the activity of SinR (a repressor of matrix gene transcription), thereby derepressing the *tapA–sipW–tasA* and *epsA–O* operons that control biofilm formation (100). Ultimately, Spo0A∼P directly or indirectly regulates the transcription of multiple genes involved in biofilm formation, sporulation, and secondary metabolite synthesis(99) (Fig. 1). Perception of potassium leakage signals by the membrane histidine kinase KinC involves its PAS–PAC domain (101).

#### Effects on target bacterial biofilms

Surfactin can regulate the activity of autoinducer-2, a key signaling molecule in quorum sensing systems that is catalytically synthesized by the LuxS protein in bacteria. By interfering with the quorum sensing mechanism in *S. aureus*, which regulates gene expression based on cell density, surfactin affects the biofilm formation process by *S. aureus* (95). In addition, volatile organic compounds produced by bacteria can facilitate intracellular or intercellular communication (97).

## EFFECTS OF BIOFILM AND QUORUM SENSING ON ANTIMICROBIAL SECONDARY METABOLITES

### Interactions between biofilms and quorumsensing

Biofilms and quorum sensing are closely interconnected. Biofilms provide a unique microenvironment for the transmission of quorum sensing signals. As a spatially structured microbial community, the function of a biofilm relies on complex symbiotic cooperation. In natural biofilms, EPS can firmly anchor cells to colonized surfaces, resisting mechanical and chemical forces (102). In addition, EPS promotes close proximity between cells, increasing the microbial cell density, thereby enhancing chemical signal communication among cells (103) and facilitating increases in the concentrations of autoinducing peptides (AIPs) secreted by bacteria. AIPs can activate the quorum sensing system when they reach a specific threshold concentration.

Conversely, quorum sensing, as a bacterial communication system, can also influence the formation and stability of biofilms (104). During the maturation stage of biofilm formation, quorum sensing is triggered when the cell density reaches a threshold, activating intercellular chemical communication processes, synergistically inducing bacteria to transition from a relatively independent, free-living state to a biofilm-based lifestyle (105). Moreover, some genes activated by the quorum sensing system are involved in the synthesis and regulation of biofilm-related substances, altering bacterial morphology, and releasing protective substances to resist bacteriophages, thereby maintaining biofilm stability (106). Quorum sensing affects cell differentiation and gene expression by regulating factors such as Spo0A, DegU, and ComA. The ComX quorum sensing system promotes the phosphorylation of Spo0A by enhancing the synthesis of the lipopeptide surfactin, thereby stimulating the production of biofilm matrix and driving biofilm formation (17). Furthermore, quorum sensing influences other extracellular products in biofilms, such as the synthesis of extracellular proteases. The signaling peptide ComX is sensitive to degradation by extracellular proteases, which adds complexity to the regulation of extracellular product generation (17). When the signaling molecule surfactin induces potassium leakage, the PAS–PAC domain of the membrane protein kinase KinC can sense this potassium leakage signal and induce the expression of genes involved in the synthesis of biofilm extracellular matrix (101). *P. aeruginosa* cells lacking the LAS–QS system form abnormally structured biofilms with a flattened morphology. This structural alteration increases the sensitivity of bacteria within the biofilm to antibiotic treatment, indicating that the LAS–QS system is crucial for the formation of normal biofilm structure and further affects bacterial resistance to antibiotics, reflecting the significant impacts of the quorum sensing system on biofilm properties (107).

### Synergistic effects of biofilm and quorumsensing on antimicrobialsecondarymetabolites

**Fig 2:**
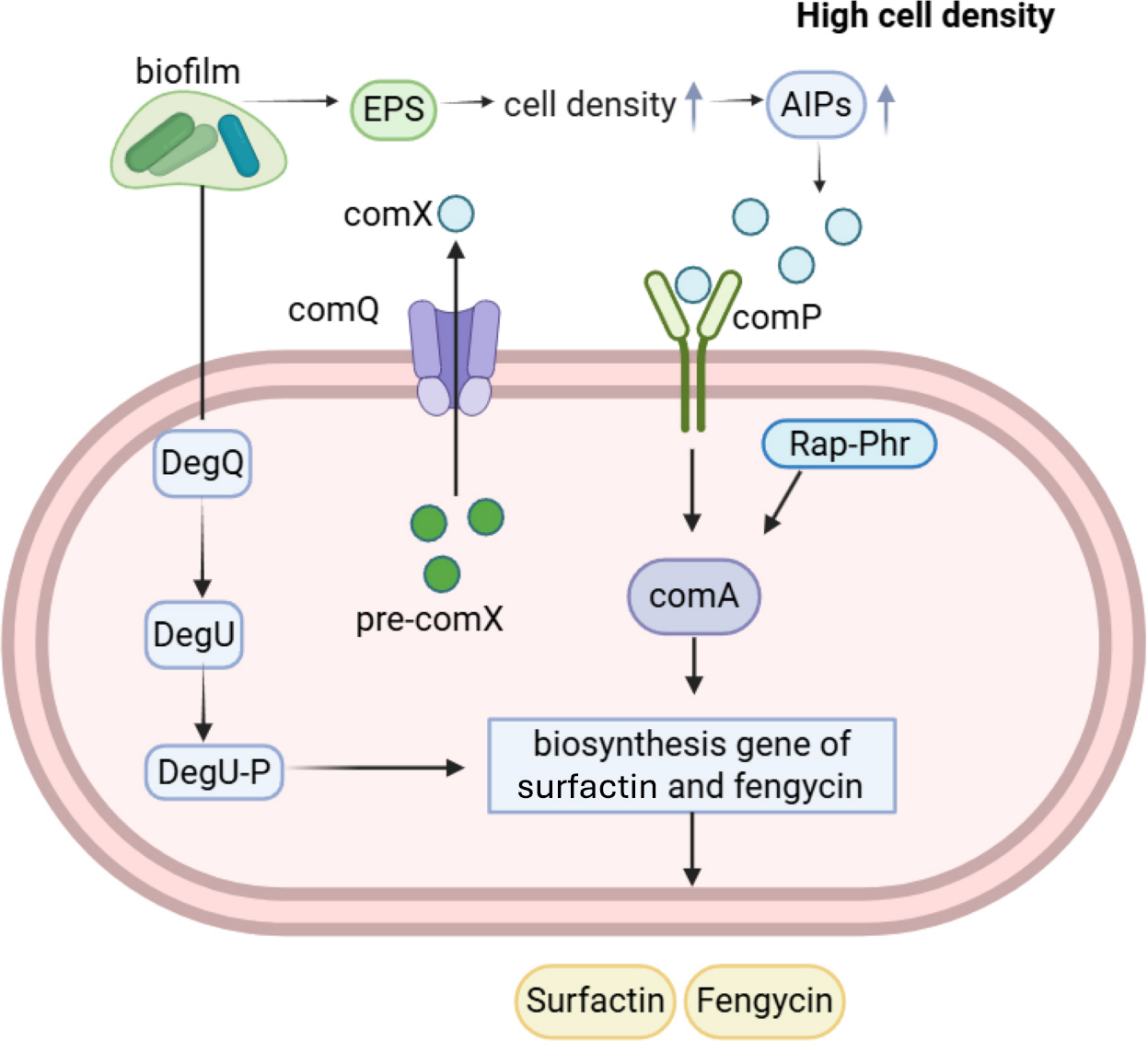
Mechanisms that allow biofilm and quorum sensing to regulate secondary metabolite production. Intracellular precursor Pre-ComX is processed by ComQ and exported as mature ComX. As the bacterial density increases within the biofilm matrix due to extracellular polymeric substances (EPS), the concentration of AIPs rises. Extracellular ComX accumulates to a threshold concentration, activating the ComP/ComA two-component signaling pathway. Concurrently, the Rap*–*Phr system modulates the activity of ComA, enabling ComA to bind to promoter regions of genes responsible for surfactin and fengycin syntheses, thereby promoting their transcription.

Biofilms and quorum sensing have a synergistic effect on regulating the antimicrobial secondary metabolites of *B. subtilis*. The ComX oligopeptide and ComP/ComA two-component system act together to regulate behaviors such as antibiotic production and the secretion of degrading enzymes (108). For instance, they regulate the expression of the *srfA* operon, which supports the synthesis of key lipopeptides such as surfactin (31), and alter the levels of DegQ, which modulates surfactin synthesis and extracellular protease production (84). They can also regulate the biosynthesis of difficidin by controlling the phosphorylation level of Spo0A (99). The Rap–Phr system is also involved by indirectly influencing the production of secondary metabolites through regulating the activity of ComA (Fig. 2).

#### Regulation of antimicrobialsecondarymetabolites by biofilmformation

The complex structure of a biofilm can create a stable living environment for internal microorganisms and reduce external interference (109). Biofilm formation is beneficial for bacterial survival, enhances their drug resistance, promotes bacterial proliferation, and stimulates the production of antimicrobial secondary metabolites (110). For example, when *B. subtilis* forms a biofilm, its cells secrete various substances, including EPS, BslA, TapA, TasA, and fengycin, which collectively constitute the extracellular matrix. The components of the extracellular matrix can affect the production and function of antimicrobial secondary metabolites. TasA and fengycin can act on seed oil bodies, triggering changes in plant lipid metabolism and glutathione-related molecule accumulation, thereby influencing the effects of antimicrobial secondary metabolites in plants (111).

Moreover, in the early stage of *B. subtilis* biofilm formation, some regulatory genes related to population metabolism are activated. These regulatory factors can bind to the promoter regions of gene clusters involved in antimicrobial secondary metabolite synthesis to promote transcription and thus increase the yields of antimicrobial metabolites. For example, two transcription factors associated with biofilm synthesis, DegQ and DegU, have significant impacts on lipopeptide production (112). The upregulated expression of the *srfA* (surfactin synthetase gene) operon, which is responsible for surfactin synthesis, leads to a substantial increase in surfactin production(113) (Fig. 2). In the iturin A production process, significant differences in biofilm formation and the iturin A yield are observed under different culture conditions and component compositions, indicating that supplementing specific components may improve the biofilm growth status, thereby increasing the yield of iturin A (109).

#### Regulation of antimicrobialsecondarymetabolites by quorumsensingsystems

Quorum sensing systems play significant roles in regulating the synthesis of antimicrobial secondary metabolites by *B. subtilis*. For example, quorum sensing signals are weak when the cell density of *B. subtilis* is low, and the expression of fengycin synthesis genes is inhibited. As the bacterial density increases, the concentration of AIPs rises, activating the ComP/ComA signaling pathway. The activated ComA protein acts as a transcription factor and binds to the promoter region of the fengycin synthesis gene cluster, promoting gene transcription, and thus increasing fengycin production. Studies have shown that at a high cell density, the expression levels of genes related to fengycin synthesis are significantly upregulated under control by the quorum sensing system. When the cell density of *B. subtilis* is high, the ComX signaling molecule triggers a series of ComA-regulated processes, such as competence development and surfactin production(114) (Fig. 2). In addition, AIPs regulate the production of bacteriocins through quorum sensing (115).

Furthermore, under regulation by quorum sensing, the antimicrobial secondary metabolites produced by *B. subtilis* exhibit unique mechanisms of action against pathogenic bacteria. Fengycin and subtilin are synthesized in large quantities under regulation by quorum sensing, and they can inhibit the growth of pathogenic bacteria through multiple pathways. First, they can disrupt the integrity of the cell membrane in pathogenic bacteria in a similar manner to surfactin, but with different molecular targets. Second, they can interfere with cell wall synthesis in pathogenic bacteria. For example, subtilin can inhibit the synthesis of important polysaccharides such as chitin, glucan, and cellulose in fungal cell walls, as well as accelerating the decomposition of these polysaccharides (116). Subtilin can also specifically bind to the precursor peptidoglycan in bacterial cell walls, interfering with the cell wall synthesis process (117).

#### Effects of biofilmstriggered by quorumsensing on antimicrobialsecondarymetabolites

Quorum sensing not only directly affects the production of antimicrobial secondary metabolites but also indirectly regulates them by triggering biofilm formation. When quorum sensing signals reach a certain threshold, they influence the phosphorylation of Spo0A, initiating a series of gene expression programs that induce *B. subtilis* to transition from a planktonic state to a biofilm state (118). During this process, the expression of biofilm-related genes and antimicrobial secondary metabolic genes is regulated in a coordinated manner. In particular, when the quorum sensing signal concentration reaches the threshold, biofilm formation is triggered and the physical barrier formed by the biofilm matrix reduces the permeability of antimicrobial secondary metabolites (119). In addition, the newly formed biofilm microenvironment can further optimize the synthesis conditions for antimicrobial secondary metabolites by regulating the distribution of nutrients and metabolic flux, synergistically enhancing the stress resistance effect of the biofilm (120). Moreover, the transmission of quorum sensing signals is more complex and efficient within the biofilm, potentially triggering the development of a more refined regulatory network for antimicrobial secondary metabolism. A quorum sensing signal concentration gradient exists within the biofilm, with higher concentrations in the inner layer and lower concentrations in the outer layer (121). This local difference in the quorum sensing signal concentration within the biofilm leads to the localization of antimicrobial metabolite synthesis (122).

## APPLICATIONS OF ANTIMICROBIAL SECONDARY METABOLITES REGULATED BY BIOFILM AND QUORUM SENSING MECHANISMS

### Applications in the foodindustry

#### Food preservation and antisepsis

**Fig 3:**
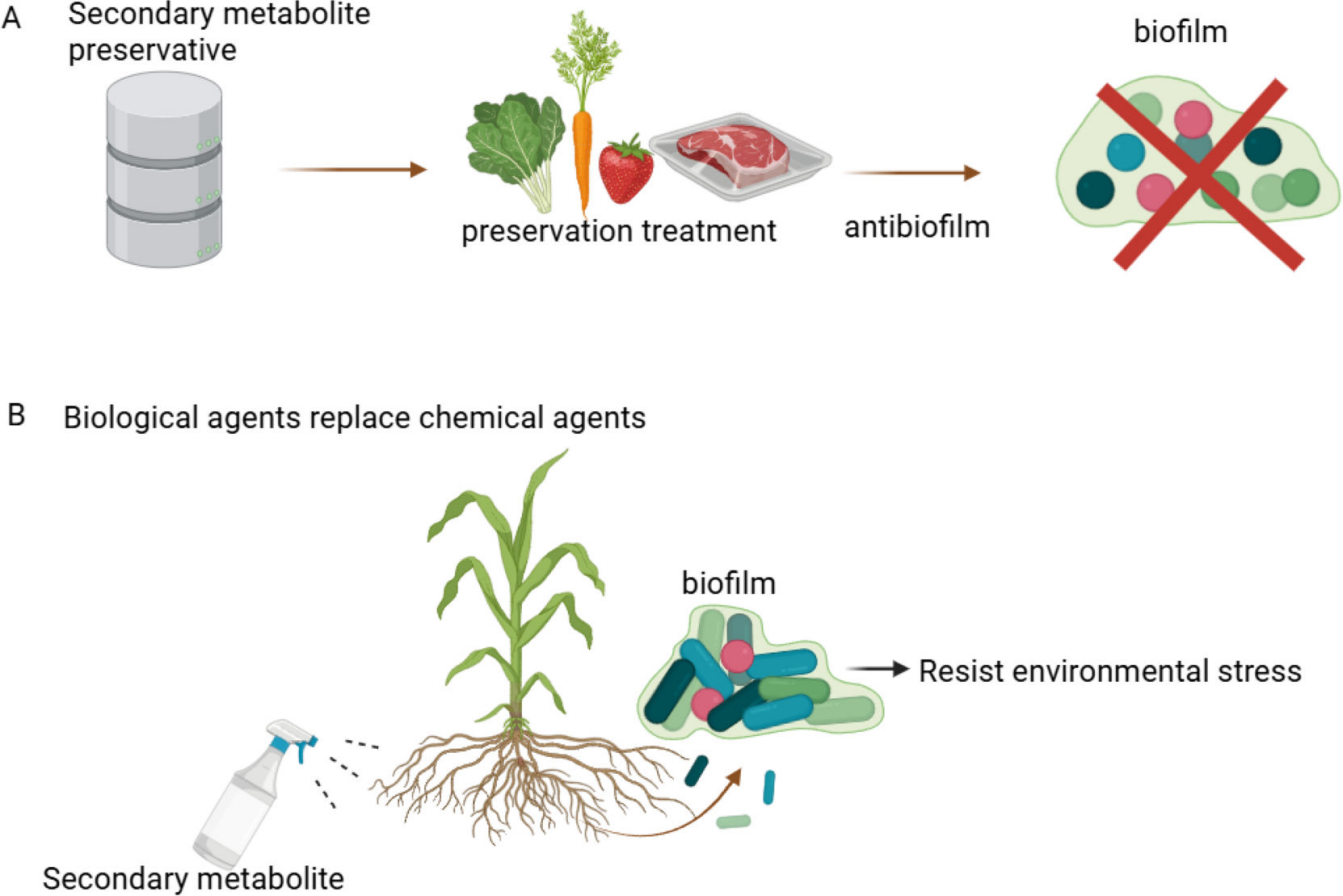
Applications of secondary metabolites based on biofilm and quorum sensing. (**A**) Secondary metabolites of *B. subtilis* act on food products, inhibiting the attachment of biofilms to food surfaces. (**B**) Biological agent pesticides containing secondary metabolites of *B. subtilis* can act in the plant rhizosphere, forming biofilms to prevent and control plant diseases.

*B. subtilis* is recognized as a safe strain by the US Food and Drug Administration, and its secondary metabolites can be used for food preservation to extend the shelf life of food products. Studies have shown that the lipopeptide surfactin can be applied in biofilm control, reducing the adhesion of pathogenic bacteria to polypropylene-contacting foods (123). Bacteriocins exhibit bactericidal activity and significantly inhibit most clinically isolated strains of *S. aureus* and *S. epidermidis* (124). Various bacteriocin-producing bacteria, including some *B. subtilis* strains, have been isolated from mushroom substrates. The cell-free supernatant containing subtilin obtained from these strains can significantly inhibit biofilm formation by *Listeria monocytogenes* on polystyrene and stainless steel surfaces (125). In meat preservation, surfactin can inhibit the growth of microorganisms on meat surfaces, maintaining the freshness and quality of meat products (Fig. 3A). Treatment with lipopeptides reduces the colony count of *Aeromonas veronii*, resulting in sparse biofilm structures in a dose-dependent manner, effectively reducing the adhesion and survival of *Aeromonas veronii* on fish meat surfaces and lowering the risk of contamination (126). *S. aureus* is a major contaminating microorganism in food supplements because it produces enterotoxins that cause numerous foodborne diseases. Surfactin can effectively inhibit the growth of *S. aureus* and is used for pork preservation. Surfactin has been shown to induce the deformation of cell morphology, disrupt the integrity of the cell wall and cell membrane, and reduce the metabolic activity of *S. aureus*, collectively contributing to the inhibition of bacterial growth and ultimately leading to cell death (33). In postharvest fruit preservation, surfactin can replace chemical fungicides to prevent or control postharvest gray mold in fruits and vegetables (127). Fengycin can induce apoptosis and necrosis of rhizobia, which symbiotically fix nitrogen with legumes, promoting plant growth, enhancing disease resistance and stress tolerance, and reducing the chance of pathogenic infection, and thus make agricultural products more storable and reduce the risk of food spoilage caused by diseases, highlighting the potential application of fengycin in food antisepsis. The antifungal activity of fengycin can be primarily attributed to its ability to disrupt the integrity of the cell membrane, interfere with the quorum sensing system, and induce cellular apoptosis (128). Moreover, due to advantages in terms of the low toxicity and excellent antibacterial activity of surfactin produced by microbial fermentation (129), adding *B. subtilis* fermentation broth containing surfactin and other secondary metabolites to food can inhibit the growth of harmful microorganisms in food and reduce food spoilage. Due to the advantages of iturin, such as safety in humans and animals, broad antibacterial spectrum, strong inhibitory effect, and low risk of inducing drug resistance, it has shown significant efficacy in the biological control of postharvest fungal diseases of fruits and vegetables. Iturin was confirmed to be effective against various postharvest diseases such as strawberry gray mold and mango anthracnose. Furthermore, advancements in fermentation technology have significantly increased iturin production (130).

#### Applications in foodprocessing

During food production, metabolites of *B. subtilis* can be used as food additives to improve the texture of food and enhance flavor. For example, lipopeptide bioemulsifiers produced by *B. subtilis* significantly optimize the texture of bread and enhance the resistance of bread to microbial proliferation (131). Spore-deficient *B. subtilis* strains such as 3NA, 168 S, and PY79S can produce diacetyl, a flavor compound that imparts a buttery aroma, which is widely used in the food and beverage industry (12). *B. subtilis* is also an important microorganism in various fermented foods, such as fermented soybeans (132) and yogurt (133). During food fermentation, secondary metabolites of *B. subtilis* can inhibit contamination by various bacteria, ensuring the smooth progress of the fermentation process. Mycosubtilin isolated and purified from *B. subtilis* BS-Z15 fermentation broth inhibits *Aspergillus flavus* in peanuts, significantly suppressing *A.flavus* infection and toxin production. The inhibitory effect of mycosubtilin on *A. flavus* is associated with its regulation of genes related to membrane transporters, cell wall biosynthesis, transcription and translation processes, and toxin synthesis (134). In addition, *B. subtilis* MB40 strain, which is applied as a dietary supplement for human consumption, has undergone a series of tests for enterotoxins and antibiotic resistance, ensuring the safety of *B. subtilis* used in food produced for human health purposes (135).

### Applications in agriculture

#### Plant diseasecontrol

The utilization of antimicrobial secondary metabolites from *B. subtilis* is a green and eco-friendly strategy for plant disease control. The biofilm structures of *B. subtilis* can colonize the plant rhizosphere and release various antimicrobial secondary metabolites (such as lipopeptides and polyketide antibiotics) through quorum sensing regulation (Fig. 3B). These antimicrobial compounds can effectively inhibit growth and infection by plant pathogens, with significant inhibitory effects on soilborne pathogens such as *Fusarium oxysporum*, *Aspergillus niger*, and *Alternaria alternata* (136). For example, it has been reported that surfactin exerts its antifungal activity by inducing ROS-mediated mitochondrial apoptosis in the hyphae of *Fusarium graminearum* (137). Studies have shown that stable colonization by *B. subtilis* biofilm in the plant rhizosphere can inhibit pathogen growth through spatial competition and nutrient deprivation, as well as continuously releasing antimicrobial secondary metabolites, forming a “chemical barrier” that enhances antimicrobial persistence and provides long-term protection for plants (138). Various secondary metabolites released centrally by biofilm colonization can also achieve disease control through direct antimicrobial effects and induce plant resistance (139). In addition, *B. subtilis* can target seed oil bodies through biofilm matrix components, such as the amyloid protein TasA and lipopeptide fengycin, regulating plant metabolism and triggering systemic plant defense responses, thereby promoting plant growth and enhancing the antifungal capacity (111).

#### Development of newagriculturalbiologicalagents

*B. subtilis*-based biological agents exhibit good stability due to the rapid growth of *B. subtilis*, simple nutritional requirements, and the ability to produce heat-resistant and stress-tolerant spores. These biological agents, formulated from secondary metabolites, also possess green and safe characteristics, where they are non-toxic, harmless, and free of pesticide residues, meeting the standards for organic agriculture and green food production. Their application in agricultural production can not only reduce the use of chemical pesticides but also improve the soil quality and enhance the yields and quality of crops (140). For example, biological agents made from lipopeptides produced by *B. subtilis* can enhance the soil’s ability to degrade pollutants such as petroleum hydrocarbons, while those made from volatile organic compounds (e.g., 2-ethylhexanol and tetrahydrofuran-3-ol) can upregulate the activity of plant photosynthesis-related genes and endogenous auxin levels, promoting plant growth (141).

### Applications in aquaculture

Secondary metabolites of *B. subtilis*, such as surfactin and subtilin, can be used as feed additives or water improvers to reduce the risk of infection in aquatic animals by disrupting the cell membrane integrity in pathogens (142). As a probiotic microorganism, *B. subtilis* can secrete various extracellular enzymes, including proteases, amylases, and lipases, which enhance the digestion and assimilation of nutrients in aquaculture feeds, thereby improving feed conversion efficiency and promoting animal growth. After adding *B. subtilis*, plant-based raw materials such as soybeans, corn, and wheat can partially replace fish meal and fish oil, thereby reducing the production cost and reducing the reliance on marine resources (143). *B. subtilis* can also form biofilms to stably colonize aquaculture systems and inhibit growth and biofilm formation by pathogens in aquatic environments. Studies have shown that some lipopeptide metabolites can enhance the immune barrier in fish and improve their disease resistance. Lipopeptides and degrading enzymes secreted by *B. subtilis* biofilms can decompose residual feed, feces, and algal toxins in aquaculture water, reduce the chemical oxygen demand and ammonia nitrogen levels, and effectively improve the water quality. They can also promote colonization by beneficial bacteria such as *Lactobacillus* through quorum sensing competition, inhibit reproduction by harmful bacteria, and maintain the microecological balance of water bodies (144). Marine *B. subtilis* strain C3 can form a thin biofilm on glass surfaces or gas–liquid interfaces, inhibiting adhesion by *Vibrio harveyi*. Moreover, the secretion of surfactin can interfere with adhesion by *V. harveyi*, promote the dispersion of its mature biofilm, and prevent colonization and aggregation by *V. harveyi* (145).

## CONCLUSIONS AND PROSPECTS

**Fig 4:**
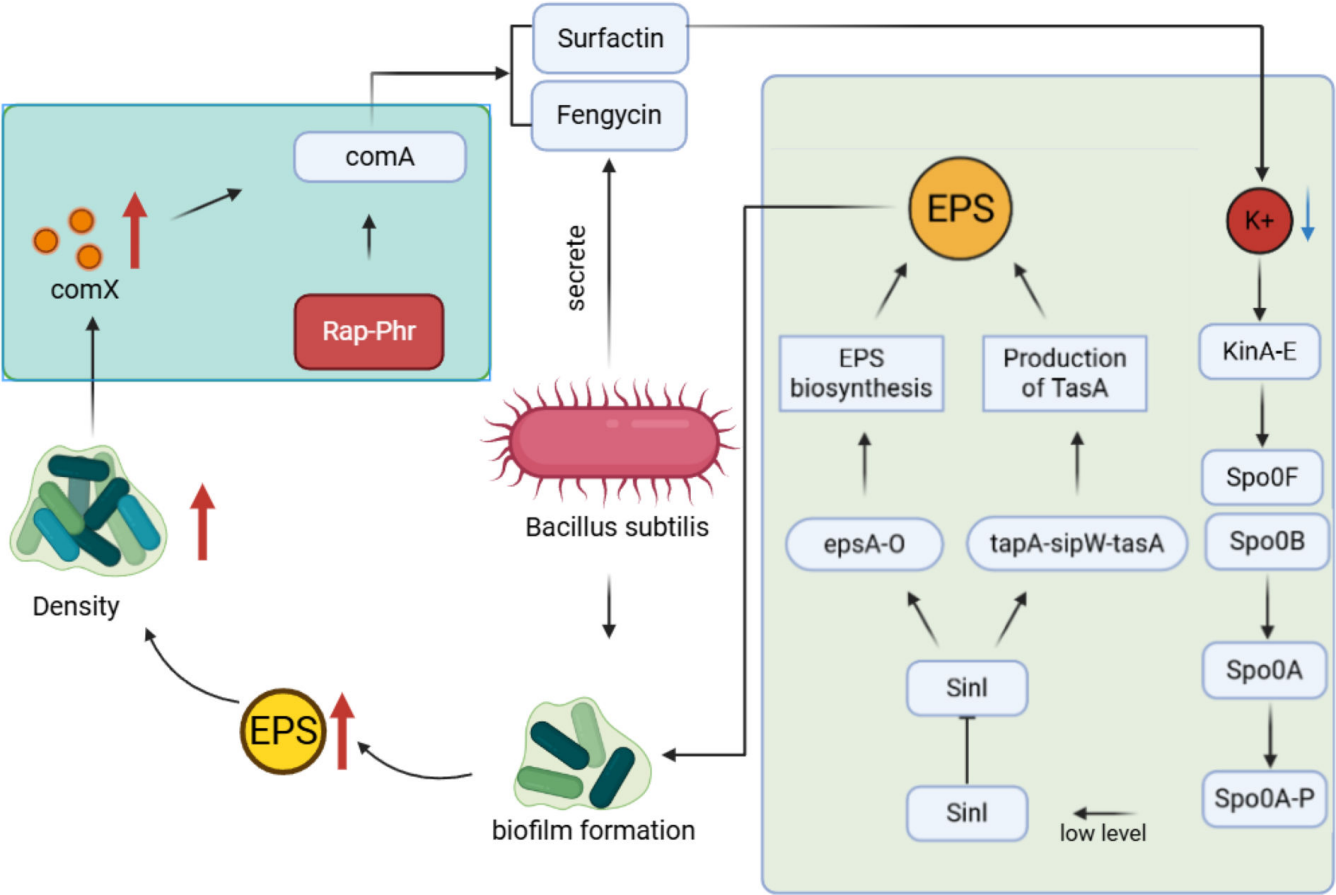
An integrated regulatory model illustrating the overall relationship about biofilm formation, quorum sensing, and antimicrobial metabolites. As cell density increases, the signaling peptide ComX activates the response regulator ComA through the quorum-sensing system, thereby promoting the production of lipopeptides such as surfactin and fengycin. Surfactin can trigger potassium ion leakage, which is sensed by histidine kinases KinA–E and activates the phosphorelay pathway (Spo0F–Spo0B–Spo0A), leading to the formation of Spo0A-P. At low levels, Spo0A-P induces SinI, which antagonizes the repressor SinR and allows the expression of the epsA–O and tapA–sipW–tasA operons. These operons direct the synthesis of extracellular polymeric substances (EPS) and TasA fibers, respectively, ultimately promoting biofilm formation.

The secondary metabolites produced by *B. subtilis* are characterized by diverse antimicrobial mechanisms, and their production and antimicrobial functions are closely associated withbiofilm formation and quorum sensing systems of *B. subtilis* (Fig. 4). The biofilm and quorum sensing systems of *B. subtilis* regulate the production of antimicrobial secondary metabolites at multiple levels, and their coordination allows these metabolites to combat pathogens through various mechanisms, collectively forming an efficient and complex antimicrobial system. Further research into biofilms and quorum sensing systems will facilitate a deeper understanding of the antimicrobial properties of *B. subtilis*, enabling better regulation of secondary metabolite production andenhanced antimicrobial efficacy. Furthermore, these secondary metabolites have significant application value in the food and agricultural sectors, providing new insights and methods for food preservation, processing, safety testing, plant disease control, and the development of novel agricultural biological agents. Future research could explore the mechanisms of action of secondary metabolites and discover more novel antimicrobial secondary metabolites. In addition, the practical application of *B. subtilis* secondary metabolites in food production and agricultural cultivation requires addressing several technical and safety challenges, such as the large-scale production of secondary metabolites, their stability, and potential impacts on human health. Based on comprehensive research and technological innovation, the applications of *B. subtilis* secondary metabolites in the food and agricultural fields are expected to be expanded further, making greater contributions to ensuring food safety and promoting the development of the food industry and agriculture.
